# Better Offensive Strategy in Basketball: A Two-Point or a Three-Point Shot?

**DOI:** 10.2478/hukin-2022-0061

**Published:** 2022-09-08

**Authors:** Huancheng Gou, Hui Zhang

**Affiliations:** 1 Department of Sport Science, College of Education, Zhejiang University, Hangzhou, China

**Keywords:** basketball, NBA, offense strategy, outside offense, inside offense

## Abstract

To better understand and explore the development trend of the offensive strategies of the world’s top basketball leagues, this study took NBA shooting data in the regular seasons from 2009/2010 to 2018/2019 as the samples and analysed the relationships between the shooting score ratio and game win probability, and the practical application of offensive strategies in games. The results showed that (1) increasing the number and the percentage of three-point offenses in the game can improve the probability of winning. However, too many two-point shots can affect the team’s winning probability to a certain extent. (2) The strong teams in the NBA focused more on the outside offense, while the weak teams focused more on the inside offense. (3) Statistical data further showed that whether a team’s opponent is strong or weak, taking the offensive strategy that tends toward the outside shot can lead to a higher game win probability than the offensive strategy that tends toward the inside offense.

## Introduction

After more than one hundred years of development, basketball has become an extremely attractive international sport. The NBA is the top basketball league in the world, and its technical and tactical development has always been a world leader. In recent years, great changes have taken place in the offensive strategies of NBA games. These changes not only affect NBA players and coaches, but also have a great impact on the development of basketball in other countries.

Shooting is the only way to score in a basketball game. Non-free throw shooting is the main scoring method in basketball games and one of the most important technical elements of the game (Hay, 1993). Compared with the low risk and stable returns of two-point field goals, the high returns of outside three-point shots require the team to take higher risks. In the game, an offense which mainly relies on the mid-range two-point shot close to the basket is called an "inside game", and the use of a large number of long-distance three-point shots is called an “outside game” (George et al., 2009). In professional basketball leagues, two- and three-point field goal scores have an important effect on team wins and rankings (García et al., 2013; Gomez et al., 2016; Puente et al., 2015). The success of elite basketball teams seems to depend not on how many opportunities the team has to score, but on how the team benefits from the current scoring opportunities (Ibez et al., 2008). During the basketball game, choosing a reasonable shooting (offensive) strategy is of great significance to the team’s winning.

At present, the research on basketball games mainly focuses on the players’ technical performance and the outcome of the game. Researchers have studied a large amount of competition data of various national basketball leagues and tournaments, obtaining valuable results, and have found that different leagues have different key techniques. For example, during the 2003-2011 seasons, the key factors affecting team strength in the NBA were winning probability, offensive efficiency, third-quarter scores, steals and turnovers per game (Mikołajec et al., 2013). In the NBA’s balanced game, defensive rebounds, blocks, and assists determine the outcome of a strong team, while defensive rebounds and turnovers are key performance indicators for weak teams (Zhang et al., 2019).

The data of the Spanish Professional Basketball League (ACB League) in the 2003-2013 seasons show that the field goal percentage is a key factor affecting the winning probability in elite basketball games (Puente et al., 2015). In the ACB league matches where the strength of the two sides is balanced, the winner performs better in assists and two-point field goals, while the winner of an unbalanced game has more three-point field goals (García et al., 2014). In Euroleague Basketball, two-point and three-point field goals, steals, and defensive rebounds are the key elements of a balanced game. In an unbalanced game, two-point field goals and defensive rebounds are important factors for winning (Çene, 2018). Free throws and forcing opposing players to foul in World Championships play an important role in the team’s victory (Malarranha et al., 2013). Assists, two-point and three-point field goals were significant for winning in the Basketball World Cup 2019, and two-point field goal attempts were negatively correlated with team strength while three-point field goal attempts displayed a positive relationship with it (Dong et al., 2021; Stavropoulos et al., 2021). The above studies mainly distinguish between multiple variables of technical and tactical performance according to different game results (wins or losses or point differences) and screen the key performance indicators of the game.

Sports performance analysis research aims to determine key performance indicators and coaches’ effective offensive strategies. The accumulation of data from a large number of games can identify a team’s offensive and defensive patterns, and the team can prepare for the game based on the opponent’s expected offensive and defensive strategies (Lord et al., 2020). The role of tactical performance indicators is to reflect the relative importance of speed, space, physical fitness and movement, as well as how players respond to their respective technical strengths and weaknesses based on the performance of both sides, which is ultimately reflected in the combination of a team’s offensive and defensive characteristics and game behaviour (Hughes and Bartlett, 2002). In basketball games, successful shooting is the ultimate goal of tactical offense (Gryko et al., 2018). In the basketball game, the shooting score is a combination of skills and tactics. Therefore, the use of isolated personal data in the analysis of team sports cannot well reflect the overall situation of the team. Independent indicators of the game statistics are unified (or converted) into a specific indicator (such as various ratios) to help compare performance between teams (Hughes and Bartlett, 2002).

Basketball is a game of the accumulation of points. Shooting ability is the performance of the team’s strength. In a limited offensive round, the different risks and benefits of shooting restrict each other, and the ratio of shots to points can express the proportion of the team’s effective offensive strategy. In view of this, this article selects the team’s three-point and two-point shooting data to analyse the team’s tactical shooting (offensive) strategy and its relationship with the game win probability.

## Methods

### Samples

A total of 24,118 regular season games of 30 teams in 10 NBA seasons from 2009 to 2019 were selected as samples; the data were obtained from the public NBA official website (www.NBA.com/Stats) with permission, and the study was approved by the local institutional ethics committee.

### Calculation Formula of Relevant Indexes

The free throw score ratio refers to the ratio of the team’s free throw score to the total score in a game, as shown in formula (1):

Free throw score ratio = (free throw score/total score)×100% (1)

The two-point score ratio refers to the ratio of the team’s two-point field goals to the total score in a game, as shown in Formula (2):

Two-point score ratio = (two-point field goals score/total score)×100% (2)

The three-point score ratio refers to the ratio of the team’s three-point field goal score to the total score in a game, as shown in Formula (3):

Three-point score ratio = (three-point field goals score/total score)×100% (3)

Offensive tendency coefficient: The three-point scoring ratio divided by the two-point score ratio is used to indicate a team’s tendency to have an effective offense.

When the value of this coefficient is large, it indicates that the team is more inclined to engage in outside attacks; otherwise, the team is more inclined to engage in inside attacks, as shown in Formula (4):

Offensive tendency coefficient (OTC) = three-point score ratio/two-point score ratio (4)

According to the team’s offensive tendency coefficient (using the percentile method), the team’s offensive strategy can be focused on internal lines (0 ≤ OTC < 0.31, *N* = 8393), a balance between internal and external offense (0.31 ≤ OTC < 0.50, *N* = 8099) and a focus on outside lines (OTC ≥ 0.50, *N* = 7626).

### Advantage Competition and Disadvantage Competition

Games can be divided into the following six types according to the difference between the total scores of the two sides (Ferreira et al., 2014):

(1) Weak advantaged side: the total score of the winning side is greater than that of the other side 1 ≤ X < 10

(2) General advantaged side: the total score of the winning party is greater than that of the other side 10 ≤ X ≤ 15

(3) Extreme advantaged side: the total score of the winning side is greater than that of the other side X > 15

(4) Weak disadvantaged side: the total score of the losing side is less than the opponent’s -10 < X ≤ -1

(5) General disadvantaged side: the total score of the losing side is less than the opponent’s -15 ≤ X ≤ -10

(6) Extreme disadvantaged side: The total score of the losing side is less than the opponent’s X < -15

### Strong Teams and Weak Teams

The strength of a team can be divided according to its ranking in a season (Sampaio et al., 2010). According to whether a team enters the playoffs, the 30 teams in the NBA can be divided into 16 strong teams and 14 weak teams.

## Results

### The Relationship between the Shoot-To-Score Ratio, Team Offensive Tendency Coefficient and Game Win Probability

**Figure 1:**
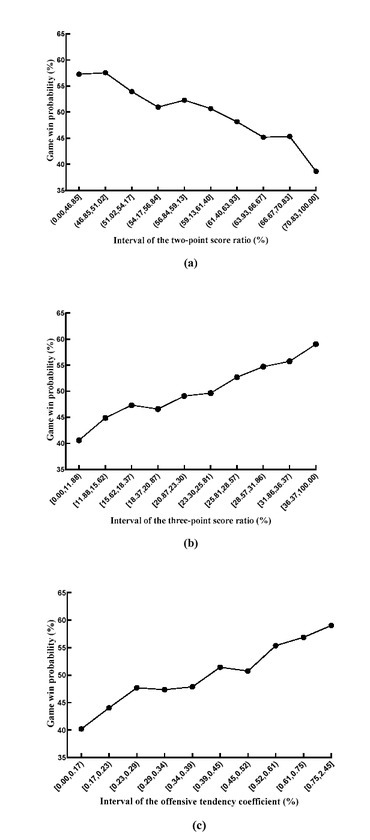
The relationship between the shoot-to-score ratio, team offensive tendency coefficient and game win probability (2009/2010-2018/2019 seasons).

Figure 1a shows the relationship between the NBA teams’ two-point score ratio (divided into 10 intervals) and the win probability. With the increase in the two-point score ratio, the team’s win probability drops significantly (showing a clear negative correlation trend). Among them, when the two-point score ratio was between 46.84 and 51.02%, the game win probability was the highest (57.55%); when the two-pointer score ratio was above 70.83%, the game win probability dropped to 38.64%.

Figure 1b shows the relationship between the NBA teams’ three-point score ratio (divided into 10 intervals) and the win probability. There was a positive correlation between the three-point score ratio and the game win probability. As the team’s three-point score ratio increased, its game win probability also gradually increased. When the three-point score ratio was higher than 36.37%, the game win probability was the highest (59.02%), and when the three-point score ratio was lower than 11.88%, the game win probability was the lowest (40.59%).

Figure 1c shows the relationship between the NBA teams’ offensive tendency coefficient (divided into 10 intervals) and game win probability. The team’s offensive tendency coefficient and the game win probability also showed a positive correlation trend. When the team’s offensive tendency coefficient increased (that is, the team was more inclined to adopt outside offensive strategies), its game win probability also gradually increased. For example, when a team’s offensive tendency coefficient was below 0.17, the team’s win probability was only 40.21%. In contrast, when the team’s offensive tendency coefficient was above 0.75, the team’s win probability in the game rose to 59.05%; that is, the team’s win probability increased by approximately 20%.

### The Score Ratio and the Offensive Tendency Coefficient of Advantaged and Disadvantaged Competitions

Table 1 shows the statistical analysis of the two-point score ratio, the three-point score ratio and the offensive tendency coefficient in the different games. In general, there were significant differences in the two-point score ratio (*F* = 72.829, *p* < 0.01, *η*^2^ = 0.013), the three-point score ratio (*F* = 137.800, *p* < 0.01, *η*^2^ = 0.022), and the offensive tendency coefficients (*F* = 104.500, *p* < 0.01, *η*^2^ = 0.018) between the advantaged and disadvantaged competitions. In terms of the two-point score ratio, the losing side’s two-point score ratio was higher than that of the winning side. The extremely advantaged side’s two-point score ratio was 57.38%, which was significantly lower than that of the weakly disadvantaged side, the generally disadvantaged side and the extremely disadvantaged side (*p* < 0.01). The two-point score ratios of the dominant side (extremely, generally, weakly) were also significantly different (*p* < 0.01), and the two-point score ratios of the weakly disadvantaged side were significantly lower than those of the generally disadvantaged side and the extremely disadvantaged side (*p* < 0.01).

**Table 1:** The score ratios and offensive tendency coefficients in different games.

	*N*	Two-point score ratio	Three-point score ratio	Offensive tendency coefficient
Extremely advantage	2953	57.38±9.34^A^	27.23±9.62^A^	0.51±0.27^A^
General advantage	2791	58.52±9.23^B^	24.82±9.42^B^	0.46±0.25^B^
Weak advantage	6315	57.95±9.17^C^	23.69±9.29^C^	0.44±0.25^BC^
Weak disadvantage	6315	59.37±9.35^D^	23.70±9.42^C^	0.43±0.24^C^
General disadvantage	2791	60.45±9.38^E^	22.29±9.41^D^	0.40±0.24^D^
Extremely disadvantage	2953	60.78±9.26^E^	21.45±9.12^E^	0.38±0.22^d^
*F*		72.829	137.800	104.500
*P*		<0.01	<0.01	<0.01
*η* ^2^		0.013	0.022	0.018

*Note: The letters are arranged in order of average size. The two groups of indicators have the same letter and the same capitalization, indicating that there is no difference between the groups (p > 0.05). There are the same letters but different capitalization, indicating that there is a difference between the groups (p < 0.05). Without the same letter, it means that there is a significant difference between the groups (p < 0.01)*.

In terms of the three-point scoring ratio, the extremely advantaged side had the highest three-point score ratio (27.23%), which was significantly higher than that of the generally advantaged side. The generally advantaged side was second (24.82%), with a significantly higher score ratio than the other groups. There was no significant difference in the three-point score ratio between the weakly advantaged side and the weakly disadvantaged side, but both sides had significantly higher score ratios than the generally disadvantaged side and the extremely disadvantaged side.

In terms of the team’s offensive tendency coefficient, the extremely advantaged side had the highest offensive tendency coefficient (0.51), which was significantly higher than that of the other groups. The offensive tendency coefficient of the generally advantaged side (0.46) was significantly higher than that of the disadvantaged side. The offensive tendency coefficient (0.44) of the weakly advantaged side was also significantly higher than that of the generally disadvantaged side and the extremely disadvantaged side.

### Analysis of Team Offensive Characteristics and Game Strategies

#### Selection of Offensive Strategies of Teams at Different Levels

**Figure 2:**
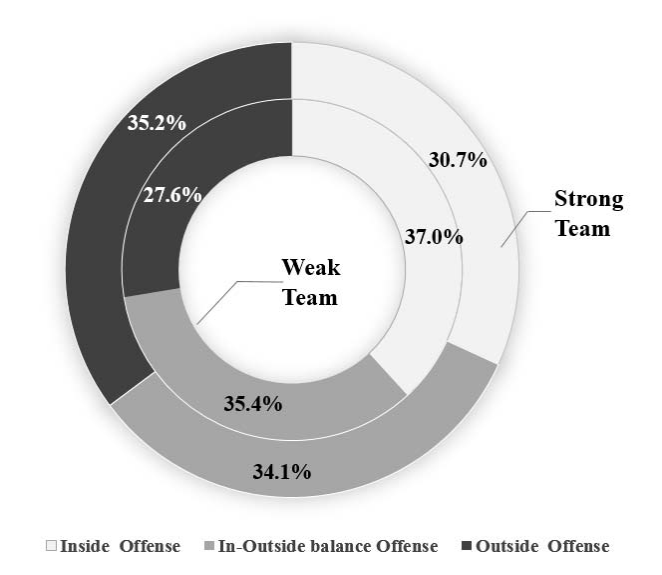
The distribution of offensive strategy selection in teams of different performance levels.

According to the team’s offensive tendency coefficient, offensive strategies were divided into three types: a focus on inside offense, a balance between inside and outside offense, and a focus on outside offense. Figure 2 shows the offensive strategies of strong teams (outer ring, *N*= 12862) and weak teams (inner ring, *N* = 11256). The three types of offensive strategies accounted for more than 30% of strong team matches, and the distribution of the three types of matches was relatively even, indicating that strong teams could flexibly and effectively change offensive strategies when facing different opponents. Among them, games that focused on outside offense (35.2%) were slightly higher than those that focused on inside offense (30.7%) and a balance between inside and outside offense (34.1%). However, weak teams tended to use more offensive strategies focused on the inside (37.0%) and a balance between inside and outside offense (35.4%) and used offensive strategies focused on the outside less often (27.6%).

### Analysis of Different Competition Strategies

**Figure 3:**
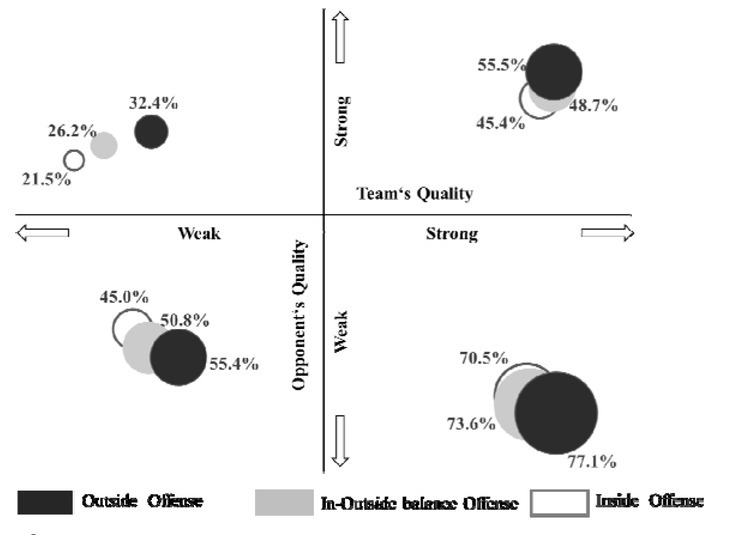
The game win probability of different offensive strategies.

The outcome of the game is not only related to the team’s own performance, but also has a significant relationship with the performance of the opponent’s competitive level. Figure 3 shows the relationship between different levels of team offensive strategies and game win probability.

In Figure 3, the horizontal axis is the strength of the team, the vertical axis is the strength of the opponent, the positive direction (to the right) of the coordinate axis is the strength of the team, and the negative direction (to the left) is the weakness of the team. Bubbles with different colours indicate different types of games. The size of the bubble represents the game win probability; the higher the win probability, the larger the bubble. The distance between the centre of the bubble and the vertical axis represents the number of games with different offensive inclination strategies, and the greater the distance, the more games adopted the strategy.

In the games between two strong teams, when the inside and outside lines of offense were balanced or when the inside line was emphasized, the team’s win probabilities were 48.7% and 45.4%, respectively; when the focus was on the outside offense, the team’s win probability reached 55.5%.

In the games between two weak teams, the team’s win probability was only 45.0% when the attack was focused on the inside; when the attack was balanced between the inside and the outside, the team’s win probability was 50.8%; and when the attack was focused on the outside, the team’s win probability reached 55.4%.

In the games pitting weak teams against strong teams, all three offensive strategies entailed a low win probability, but when the weak team adopted an offense that focused on the outside, the team’s game win probability increased to 32.4%. When the weak team focused on the inside strategies or adopted a balance of offensive strategies, the win probabilities reached only 21.5% and 26.2%, respectively. Strong teams used more outside offensive strategies, while weak teams used more inside offensive strategies.

In addition, considering the distance between the bubble centre and the vertical axis (where a bubble centre farther from the vertical axis indicates the use of the strategy in more games), strong teams focused more on outside offense, while weak teams focused on inside offense. However, for either a strong team or a weak team facing an opponent of the opposite level, an attack that focused on the inside reduced the game win probability, and the strategy of focusing on the outside attack could effectively increase the game win probability.

## Discussion

### The Development Trend of NBA Shooting Technique

From the shooting data of NBA games in the past ten seasons, the free-throw data were relatively stable, which can reflect the level of NBA referees and the stability of players’ free-throw techniques to a certain extent. Two-point field goals were always the main scoring method in games. The two-point shot data in the past three seasons show a decreasing shooting frequency and a rising shooting percentage, but generally, the two-point score ratio gradually decreased. In recent seasons, three-point field goals increased in the number of shots and showed stable shooting percentages, indicating that teams were increasingly pursuing offensive strategies aimed at three-point field goals, and the three-point score ratio gradually increased. These changes indicate that NBA teams are shifting some of the two-point offense opportunities to the three-point offense. The outside offensive strategy is becoming increasingly popular with the teams in the NBA, and it has become an important offensive method for winning the game. The coaches of the teams that make the playoffs pay more attention to players’ three-point shooting ability (Jia et al., 2018). Paying attention to three-point shooting has become a new trend in the development of professional basketball league skills and tactics and will play an important role in future basketball games.

The high percentage of three-point shooting in the world-class competitions indicates that three-point shooting has changed from a relatively difficult technique to a general technique, which has guiding significance for training and development of basketball skills and tactics globally (Tang et al., 2011).

### Competitive Strategy and Game Win Probability

The basketball concept based on “run and gun” tactics has had a profound impact on the offensive strategies of NBA games. A notable feature of the “run and gun” tactics is that the team seizes offensive space through a large number of outside shots and fast offenses (Ruan et al., 2011). The offensive characteristics of the teams in the past ten seasons can also clearly show that outside offenses are becoming increasingly common in the NBA.

The focus on the inside offensive strategy is reflected in a large number of two-pointers and fewer three-pointers. The two-point offense can keep the score rising steadily, but only after the opponent’s repeated offense fails can the team gain the advantage, which is mainly based on the team’s defensive quality. The offensive strategy that focuses on the outside is reflected in a large number of effective three-pointers. Three-pointers can increase a team’s advantage or reduce its disadvantage in scoring, and these advantages are mainly based on the quality of the team’s offense. Both shooting and defensive performance can have a strong impact on the outcome of a game. The winning team usually has better offensive and defensive performance, mainly observed in a higher three-point shooting rate under a high-pressure defence, while the losing team chooses more mid-range shooting when facing the defence (Csataljay et al., 2013).

The ultimate goal of most tactics in modern professional basketball games is to obtain offensive scoring opportunities under weak defence conditions. After obtaining the ball, launching quick offenses and long three-point shots can lead to this goal (Wu and Jia, 2018). Under the existing offensive and defensive system, compared to the defensive pressure on the inside and mid-range shooting, the offensive space of the three-point field goal entails a higher chance of scoring. Increasing the number of three-point field goals can yield more points in the same number of offensive rounds. Three-point field goals play a key role in the success of the team (Mateus et al., 2015). Playoff teams are more inclined to choose players with excellent organizational skills and shooting ability from beyond the three-point line (Zhang et al., 2018). Pure inside or outside players have gradually disappeared, and many tall players tend to play outside, relying on their physical advantages to obtain better outside scoring opportunities (Bi et al., 2011). At present, NBA inside players have a certain three-point shooting ability, and their main role is to win rebounds, make baskets and serve as the connecting hub of the inside and outside offensive and defensive tactics. Under the development trend of modern basketball, outside offenses have become an important offensive strategy for winning the game. A focus on the outside offense can provide significant advantages in current basketball and improve a team's winning probability.

However, this paper has several limitations, while also future directions of research can be established. On the one hand, this study analysed the winning probability of shooting offensive strategies in most cases based on big data, but it may deviate from the shooting offensive strategies used by some teams; on the other hand, the offensive strategies were summarized based on total shooting data which made it difficult to analyse a specific situation in the game. Therefore, the game stage (quarter, leading, or trailing situation), a combination of players, and the information of coaches should be considered for a better understanding of basketball offensive strategies in the future.

## Conclusions

With the continuous development of basketball across the world, the offensive strategies inclined to the three-point field goal have become a new trend. Adding the importance of three-point field goals and the shooting efficiency can improve the winning probability.

Having a high proportion of two-point field goals affects a team’s probability of winning to a certain extent. At present, the strong teams in the NBA focus more on the outside offense, while the weak teams focus more on the inside offense.

The statistical data further show that whether a team’s opponent is strong or weak, the strategy of focusing on the outside offense can bring a higher probability of winning than the strategy of focusing on the inside offense. Specific tactics for each game should be further detailed according to the technical and tactical characteristics of the team and its opponent.
